# *Trans*-Cinnamic Acid Increases Adiponectin and the Phosphorylation of AMP-Activated Protein Kinase through G-Protein-Coupled Receptor Signaling in 3T3-L1 Adipocytes

**DOI:** 10.3390/ijms15022906

**Published:** 2014-02-19

**Authors:** Christina Kopp, Shiva P. Singh, Petra Regenhard, Ute Müller, Helga Sauerwein, Manfred Mielenz

**Affiliations:** Institute of Animal Science, Physiology & Hygiene Unit, University of Bonn, Katzenburgweg 7-9, Bonn 53115, Germany; E-Mails: christina.kopp@uni-bonn.de (C.K.); spsingh@uni-bonn.de (S.P.S.); ute-mueller@uni-bonn.de (U.M.); sauerwein@uni-bonn.de (H.S.)

**Keywords:** *trans*-cinnamic acid, Adiponectin, 5′adenosine monophosphate-activated protein kinase, G-protein-coupled receptor 109A

## Abstract

Adiponectin and intracellular 5′adenosine monophosphate-activated protein kinase (AMPK) are important modulators of glucose and fat metabolism. Cinnamon exerts beneficial effects by improving insulin sensitivity and blood lipids, e.g., through increasing adiponectin concentrations and AMPK activation. The underlying mechanism is unknown. The G_i_/G_o_-protein-coupled receptor (GPR) 109A stimulates adiponectin secretion after binding its ligand niacin. *Trans*-cinnamic acid (*t*CA), a compound of cinnamon is another ligand. We hypothesize whether AMPK activation and adiponectin secretion by *t*CA is transmitted by GPR signaling. Differentiated 3T3-L1 cells were incubated with pertussis toxin (PTX), an inhibitor of G_i_/G_o_-protein-coupling, and treated with different *t*CA concentrations. Treatment with *t*CA increased adiponectin and the pAMPK/AMPK ratio (*p* ≤ 0.001). PTX incubation abolished the increased pAMPK/AMPK ratio and adiponectin secretion. The latter remained increased compared to controls (*p* ≤ 0.002). *t*CA treatment stimulated adiponectin secretion and AMPK activation; the inhibitory effect of PTX suggests GPR is involved in *t*CA stimulated signaling.

## 1. Introduction

Cinnamon (CN) is known to exert several beneficial effects by improving insulin sensitivity and lipid profiles. Enhanced glucose uptake and glycogen synthesis were reported after stimulation of 3T3-L1 adipocytes with hydroxychalcone, a compound of cinnamon [1]. Khan *et al*. [2] demonstrated that supplementation with cinnamon reduces fasting serum glucose and improves blood lipid profiles in patients with type 2 diabetes. In mice treated with an extract of cinnamon bark, the concentration of the adipokine adiponectin (AdipoQ) was increased [3]. Adiponectin is mainly expressed in adipocytes [4] and is important for modulating glucose and fat metabolism in insulin-sensitive tissues like skeletal muscle and liver. Adiponectin exerts its effects via binding to its receptors AdipoR1/R2 and activation of peroxisome proliferator-activated receptor α (PPARα) and 5′adenosine monophosphate-activated protein kinase (AMPK) [5]. The AMPK is a heterotrimeric kinase complex, consisting of a catalytic α subunit and regulatory β and γ subunits [6]. Multiple isoforms of these subunits have been identified [7], and the α_1_-subunit represents the predominant isoform in adipose tissue [8] as well as in cultured 3T3-L1 cells [9]. Besides AdipoQ, metabolic active hormones like leptin or insulin, and an increased cellular AMP/ATP ratio activate AMPK through phosphorylation (pAMPK) of threonine 172 in the α_1_-subunit. Huang *et al*. [10] showed in 3T3-L1 cells *in vitro*, as well as in murine adipose tissue *in vivo*, an increased AMPK activation after supplementation with cinnamaldehyde, one compound of CN. Upon activation, AMPK switches on catabolic pathways (e.g., fatty-acid oxidation and glycolysis) and inhibits anabolic processes like cholesterol, glycogen, and protein synthesis in liver and muscle. The AMPK acts as an intra-cellular energy sensor and hence improves insulin sensitivity in insulin-sensitive tissues like adipose tissue, but here the data about AMPK and its effect remain poorly distinguished [11]. The effect of various ingredients of CN on AMPK and AdipoQ is reported, but the underlying mechanism is not characterized. *Tran*s-cinnamic acid (*t*CA), another isolated compound of cinnamon, was recently identified as a ligand of the G-protein-coupled receptor (GPR) 109A [12]. The seven transmembrane GPR109A, a member of the recently deorphanized hydroxycarboxylic acid receptor family, which is also known as HCA_2_ [13], is expressed in activated macrophages and in adipocytes [14]. The binding of GPR109A agonists like niacin and its endogenous ligand β-hydroxybutyrate has been shown to activate this receptor and stimulate AdipoQ secretion in adipose tissue [15]. Therefore, we hypothesized that *trans*-cinnamic acid, as compound of CA, stimulates AdipoQ and AMPK also through G-protein-coupled receptor signaling.

To verify this hypothesis, we investigated the changes in AdipoQ secretion and the prevalence of the phosphorylated form of AMPK in differentiated 3T3-L1 adipocytes stimulated with different concentrations of the recent characterized GPR109A ligand *t*CA. To prove signaling by G-protein-coupled receptors, the adipocytes were additionally pre-incubated with pertussis toxin (PTX), an inhibitor of G_i_/G_o_ protein coupling.

## 2. Results

**Figure 1.**
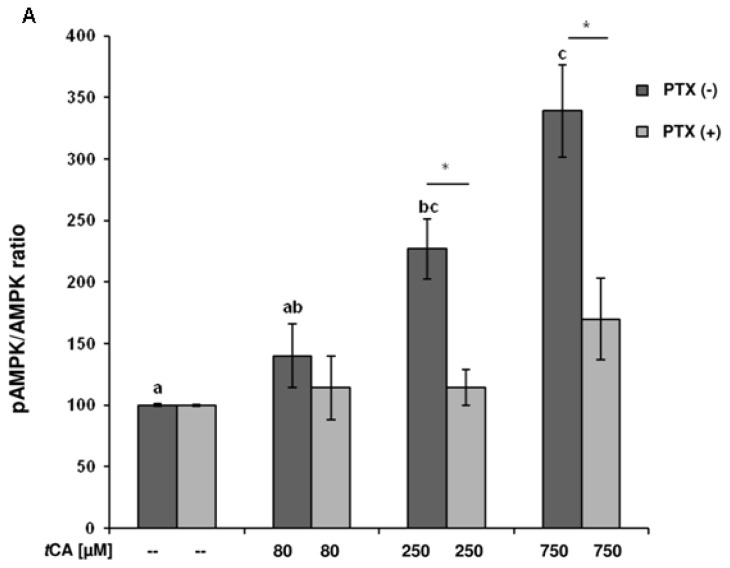

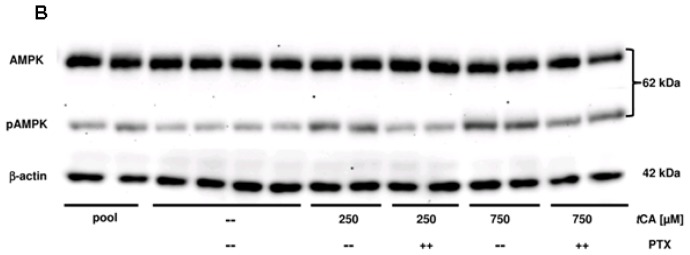
*Trans*-cinnamic acid (*t*CA) affects the intracellular 5′adenosine monophosphate-activated protein kinase (AMPK) activation by phosphorylation (pAMPK) in differentiated 3T3-L1 cells. (**A**) *t*CA effects on pAMPK/AMPK ratios in differentiated 3T3-L1 cells. After 4 h of starvation, the adipocytes were pre-incubated with (PTX (+)) or without pertussis toxin (PTX (−)) (100 ng/mL) for 16 h and then treated for 5 h with 80, 250 or 750 μM *t*CA, or with buffered saline (PBS) as controls respectively. Different lower case letters designate significant differences (*p* ≤ 0.01) between *t*CA treatments and controls. Significant differences (*p* ≤ 0.05) due to (+) or (−) PTX pre-incubation are designated with asterisks for each *t*CA treatment group. Data are expressed as means ± SEM (*n* = 6); (**B**) Representative Western blot analyses. After gel electrophoreses, membranes were incubated with specific antibodies against AMPK, pAMPK or with β-actin as loading control.

To test whether *t*CA has an effect on the phosphorylation of AMPK, the differentiated 3T3-L1 cells were stimulated with three different concentrations of *t*CA (80, 250, 750 μM) for 5 h. *t*CA acid increased (*p* ≤ 0.001) the extent of phosphorylation of Thr 172 of AMPK in a dose dependent manner (Figure 1a). When compared with the controls, activation of the AMPK was 2 and 3 times higher in cells treated with 250 or 750 μM *t*CA (*p* = 0.009 and *p* ≤ 0.001, respectively), whereas the pAMPK/AMPK ratio in 80 μM *t*CA treatment was similar to controls. To assess whether the effects of *t*CA were mediated by G_i_/G_o_-protein-coupled receptor signaling, the experiments were conducted following pre-incubation with PTX (100 ng/mL) for 16 h. Treatment with PTX dampened the increase of pAMPK/AMPK ratios after *t*CA treatment, no differences were observed neither among the treatment groups, nor in comparison to controls pre-incubated with PTX. Cells treated with 250 and 750 μM *t*CA, respectively, but without PTX pre-incubation showed two times higher pAMPK/AMPK ratios (*p* = 0.011 and *p* = 0.026) when compared to the same treatment groups with PTX incubation (Figure 1A). A representative picture of Western blot analyses is shown in Figure 1B.

**Figure 2.**
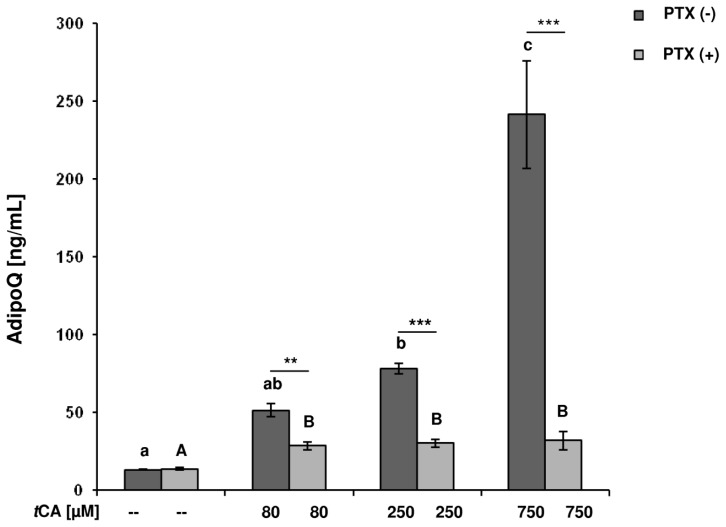
*Trans*-cinnamic acid (*t*CA) effects on AdipoQ concentrations in cell culture supernatant of differentiated 3T3-L1 cells. After 4 h of starvation, the adipocytes were pre-incubated with (PTX (+)) or without pertussis toxin (PTX (−)) (100 ng/mL) for 16 h and then treated for 5 h with 80, 250 or 750 μM *t*CA, with PBS as controls, respectively. Different lower case letters designate significant differences (*p* ≤ 0.01) between *t*CA treatments *vs*. controls for PTX (−) cells, different capital letters designate significant differences (*p* ≤ 0.01) between *t*CA treatments *vs*. controls for PTX (+) cells. Significant differences (^**^: *p* ≤ 0.01; ^***^: *p* ≤ 0.001) due to PTX (+) or PTX (−) pre-incubation for each *t*CA treatment group are indicated with asterisks. Data are expressed as means ± SEM (*n* = 6).

Treatment with *t*CA increased (*p* ≤ 0.001) AdipoQ concentrations in the cell culture supernatant dose dependently (Figure 2). When compared to controls, AdipoQ concentrations were increased five-fold (78 ± 3.4 ng/mL) after stimulation with 250 μM *t*CA (*p* = 0.005) and were about 18 times higher (241 ± 34.4 ng/mL) after treatment with 750 μM *t*CA (*p* ≤ 0.001). For all *t*CA treatment groups, pre-incubation with PTX lowered the AdipoQ concentrations to values between 28.5 ± 2.6 and 32 ± 5.9 ng/mL, but consistently higher concentrations than in the related controls (*p* ≤ 0.002) were retained.

Comparing PTX pre-incubation groups, AdipoQ concentrations decreased by 1.8 and 2.5 times after PTX pre-incubation in the 80 μM *t*CA (*p* = 0.002) and 250 *t*CA (*p* ≤ 0.001) treatment groups, respectively, compared to the corresponding PTX (−) group. In addition, the AdipoQ concentrations in the supernatant of the 750 μM treated cells decreased seven-fold (*p* ≤ 0.001) with PTX pre-incubation.

Correlation analysis across all samples confirmed a linear relationship between the AdipoQ concentrations and the pAMPK/AMPK ratio (*p* ≤ 0.001, *r* = 0.534).

## 3. Discussion

We investigated the changes in AdipoQ secretion and the prevalence of the phosphorylated form of AMPK in differentiated 3T3-L1 adipocytes stimulated with different concentrations of the recently identified GPR109A ligand *t*CA. In addition, it was to be characterized if these changes were mediated through G_i_/G_o_-protein-coupled receptor signaling. The major findings were as follows: (1) *t*CA increased secretion of AdipoQ and phosphorylation of AMPK; (2) Inhibition of GPR signaling by PTX abrogated the activating effect of *t*CA on secretion of AdipoQ and phosphorylation of AMPK but not completely. Several studies characterized CN to improve glucose and lipid profiles [1,2,16]. Various components and sources of CN were tested but we introduced *t*CA, another isolated compound, for the first time as the influencing variable on the AdipoQ system and, thereby, on glucose and fat metabolism. Corresponding to the study of Kim *et al*. [3] in which liquid Cinnamon bark extract was administered to mice, we showed that AdipoQ secretion increased in a dose dependent manner by *t*CA treatment. Adiponectin, one of the most important adipokines, improves insulin resistance and lipid metabolism [17]. The effects are mediated through its receptors AdipoR1/R2 and can at least partially be explained by their direct activation of AMPK in skeletal muscle, liver and adipose tissue [18]. Here, we showed a correlation between AdipoQ and the pAMPK/AMPK ratio, presuming an activation of AMPK subsequent to the increased secretion of AdipoQ after *t*CA treatment, supporting the study of Yamauchi *et al*. [19]. In our study, treatment with *t*CA induced phosphorylation of AMPK up to three times more than in non-treated cells. That concurs with findings of Huang *et al*. [10] where activation of AMPK after treatment with cinnamaldehyde in 3T3-L1 adipocytes was observed and confirmed by dampened effects after adding compound C, a specific inhibitor of AMPK. In addition, Huang *et al*. [10] showed in consequence of cinnamaldehyde treatment an increase in phosphorylation and, thereby, inactivation of acetyl-CoA carboxylase (ACC), which is associated with a decreased lipogenic rate and reduced lipolysis [8]. Furthermore, phosphorylation of both proteins is said to be related to increased mitochondrial fatty acid oxidation in adipose tissue [8,10]. Although the beneficial effects of CN and its compounds were proven in several studies, little is known about its signaling pathway. Ren *et al*. [12] recently identified *t*CA as a ligand of GPR109A (PUMA-G in mice), mainly expressed in immune cells and adipocytes. Niacin as another ligand of GPR109A is known to increase AdipoQ secretion [20]. Plaisance *et al*. [15] demonstrated that the AdipoQ modulating effect of niacin is mediated through the GPR109A. Mice deficient in PUMA-G (GPR109A) showed no increase in serum AdipoQ concentration after treatment with niacin. To test if the modulating effect of *t*CA on AdipoQ and AMPK is mediated by G_i_/G_o_-protein-coupled receptors, we pre-incubated the adipocytes with PTX, an inhibitor of G-protein coupling. The *t*CA-induced activation of AMPK was abolished after blocking of G_i_/G_o_ signaling, indicating this pathway is involved in the signal transmission of *t*CA. The AdipoQ secretion was, as expected, significantly decreased after receptor blocking irrespective of the *t*CA treatment group, but still significantly increased according to controls. This is in contrast to the findings of Plaisance *et al*. [15], who showed an abrogated increase in AdipoQ secretion after stimulation with niacin and PTX in rat adipocytes. Due to diminished but still higher AdipoQ concentration after PTX incubation, another possible stimulator for AdipoQ after *t*CA treatment should be discussed. Kim and Choung [3] showed an up-regulated mRNA expression of peroxisome proliferation-activated receptor (PPARγ) in adipose tissue after treatment with an extract of cinnamon bark. The transcription factor PPARγ is a known stimulator of AdipoQ expression [21] and a regulator of several genes involved in controlling insulin sensitivity [22]. Besides increased PPARγ expression, an increase of AdipoQ secretion was observed after administration of cinnamon extract, supporting this regulation as possible stimulus of AdipoQ secretion after *t*CA treatment, besides the signaling through the GPR109A [3]. The data about the expression of GPR109A and its signaling capability in 3T3-L1 adipocytes is controversial. Zhang *et al*. [23] were unable to detect gene expression of GPR109A; Jeninga *et al*. [24] showed clearly an increasing mRNA as well as protein expression of GPR109A in 3T3-L1 cells throughout differentiation, which was increased by the PPARγ agonist rosiglitazone. Also, Digby *et al*. [25] observed the expression of GPR109A mRNA, which was upregulated by TNFα. Plaisance *et al*. [15] showed protein expression of GPR109A in 3T3-L1 cells but observed no effect on AdipoQ secretion after niacin treatment, whereas Ge *et al*. [26] detected increased glycerol release after stimulation with niacin. The findings of Plaisance *et al*. [15] were annihilated when the cells were transfected with the human GPR109A orthologon HM74A. In our study, the presence of GPR109A mRNA in the differentiated 3T3-L1 *in vitro* model was proven (data not shown). The GPR109A ligand *t*CA increased the AdipoQ secretion via GPR signaling in 3T3-L1 adipocytes. Due to our experimental design, it was not possible to specify the G_i_/G_o_-protein-coupled receptors mediating the effects of *t*CA, but we assume that further studies using e.g., a specific GPR109A agonist, or primary adipocytes from GPR109A knockout mice, will define the GPR109A being involved in *t*CA signaling pathways. Furthermore, our findings after PTX pre-incubation indicate a potential, but still unknown *t*CA mediated signaling pathway besides the one through GPR signaling that might be related to the activation of PPARγ, and which should be verified in the future.

## 4. Experimental Section

### 4.1. Cell Culture

Murine 3T3-L1 fibroblast cells were seeded in 25 cm^2^ flasks at a density of 4000 cells per cm^2^ and cultured with Dulbecco’s modified eagle’s medium high glucose (DMEM) containing 10% fetal calf serum (FCS) and 10 mg/mL penicillin/streptomycin (pen/strep) (basic medium) (all from PAA, Pasching, Austria) in a humidified atmosphere of 95% air and 5% CO_2_ at 37 °C for 24 h. To induce differentiation of the 3T3-L1 fibroblasts into adipocytes, 0.5 mM 3-isobutyl-methylxanthine (IBMX) (Applichem, Darmstadt, Germany), 0.25 μM dexamethasone and 5 μg/mL bovine insulin (both from Sigma-Aldrich, St. Louis, MO, USA) were added to the basic medium for 48 h. Cells were then maintained in basic medium supplemented with 5 μg/mL bovine insulin. Media were replaced every 2 days until 85%–95% of the cells were differentiated (day 12 after initiation of differentiation), which was documented by the accumulation of lipid droplets (Oil Red O staining, 0.2%).

### 4.2. Treatment of Cells

Prior to the treatments, cells were cultured in basic medium for 24 h, then serum starved in DMEM supplemented only with 0.1% fatty acid-free bovine serum albumin (BSA) (Carl Roth, Karlsruhe, Germany) for 4 h. The adipocytes were subsequently incubated for 16 h with 100 ng/mL pertussis toxin (PTX) (Sigma-Aldrich, St. Louis, MO, USA), which selectively affects G_i_/G_o_ signaling, to characterize possible effects of *t*CA by GPR signaling. Cells were then treated with 80 μM, 240 μM or 750 μM *t*CA (Sigma-Aldrich, St. Louis, MO, USA) for 5 h (*n* = 6). Equal volumes of the solvent (phosphate buffered saline (PBS)) were applied to controls instead of PTX and *t*CA, respectively. At the end of the incubation time, supernatant was collected and stored at −20 °C until analysis. The adherent adipocytes were washed twice with ice cold PBS and lysed with pre-chilled lysis buffer as described previously [27]. The cell lysates were harvested by scraping, transferred into pre-chilled 1.5 mL tubes and centrifuged at 16,000 *g* for 20 min at 4 °C. Protein concentrations were measured according to Bradford [28].

### 4.3. Western Blot

For the detection of AMPK and pAMPK, respectively, 18 μg total protein were treated with Laemmli buffer and reduced with 4% Dithiothreitol (DTT) (Applichem, Darmstadt, Germany), boiled for 5 min at 95 °C, centrifuged for 5 min at 10,000 *g* at 4 °C, and subsequently loaded in duplicates on a 10% Mini-PROTEAN TGX Precast Gel (Bio Rad Laboratories, Munich, Germany). After electrophoresis, the fractionated proteins were transferred to a polyvinylidene difluoride (PVDF) membrane (GE Healthcare, Buckinghamshire, UK) by Trans Turbo Blot (Bio Rad Laboratories, Munich, Germany). To avoid unspecific antibody binding, the membranes were incubated in tris-buffered saline containing 0.05% Tween 20 (TBST) and 10% Rotiblock (Carl Roth, Karlsruhe, Germany) for 60 min at RT. The membranes were cut horizontally at 50–55 kDa. The upper parts of the membranes were exposed to the primary rabbit antibodies against AMPK in a dilution of 1:1000 or its phosphorylated form (pAMPK) (both 62 kDa), respectively (AMPKα, pAMPKα, Cell Signaling, Danvers, MA, USA) in a dilution of 1:500, each diluted in TBST with 5% BSA overnight at 4 °C. The lower parts of the membranes, with proteins ≤50 kDa, were incubated with a primary mouse antibody against β-actin (42 kDa) (Biovision, Milpitas, CA, USA) diluted 1:6000 in blocking solution under the same conditions. After rinsing, a horseradish peroxidase-labeled secondary anti-rabbit antibody (1:50,000; Cell Signaling, Danvers, MA, USA) or a horseradish peroxidase-labeled secondary anti-mouse antibody (1:20,000) (SouthernBiotech, Birmingham, AL, USA) were applied for 60 min at RT. Antigen-antibody immunocomplexes were revealed using enhanced chemiluminescence detection system (GE Healthcare) and densitometry analysis was performed using a Versa Doc 1000 and Image Lab software (both Bio Rad Laboratories Munich, Germany). Specific band intensities were normalized to β-actin values as an internal standard. To be able to compare the band intensities from different membranes, a 3T3-L1 pool sample was electrophoresed and blotted in duplicates on each membrane and used as reference standard. The mean intensity of the duplicate bands of the samples in relation to the mean of the standard was estimated and the ratio of pAMPK to AMPK was calculated.

### 4.4. Measurement of AdipoQ Secreted from 3T3-L1 Adipocytes

The AdipoQ content in the cell supernatant was quantified by a recently developed in-house ELISA [29] for which parallelism of mouse AdipoQ was approved. The intra- and interassay coefficients of variation were 7% and 11%, respectively.

### 4.5. Statistical Analyses

Data were analysed using IBM SPSS 20 (IBM, Ehningen, Germany) and are presented as means ± SEM. The results of the controls were not different and thus merged for further analyses, within the PTX (+) and PTX (−) treatment. For comparisons within treatment groups and between treatment and controls, ANOVA with either Bonferroni or Dunnett-T3 *post-hoc* analysis, depending on homogeneity of variances, was performed. To compare the PTX treated *versus* non PTX treated samples, data were examined using the Student’s *t*-test. Spearman-Rho correlation coefficients were calculated between the results of AdipoQ and pAMPK/AMPK. Statistical significance was declared at *p* ≤ 0.05.

## 5. Conclusions

In conclusion, treatment with *t*CA stimulated the secretion of AdipoQ and the phosphorylation of AMPK in 3T3-L1 adipocytes and therefore improves insulin sensitivity; the inhibitory effect of PTX points to a *t*CA stimulated G_i_/G_o_-protein-coupled receptor signaling pathway.
